# Developing and applying a deductive coding framework to assess the goals of Citizen/Community Jury deliberations

**DOI:** 10.1111/hex.12880

**Published:** 2019-03-12

**Authors:** Anna Mae Scott, Rebecca Sims, Chris Degeling, Stacy Carter, Rae Thomas

**Affiliations:** ^1^ Faculty of Health Sciences and Medicine, Centre for research in Evidence‐Based Practice Bond University Robina Queensland Australia; ^2^ Research for Social Change University of Wollongong Wollongong New South Wales Australia

**Keywords:** citizens jury, community jury, health, public deliberation, public engagement, quality, stakeholder involvement

## Abstract

**Background:**

Public participation in health policy decision making is thought to improve the quality of the decisions and enhance their legitimacy. Citizen/Community Juries (CJs) are a form of public participation that aims to elicit an informed community perspective on controversial topics. Reporting standards for CJ processes have already been proposed. However, less clarity exists about the standards for what constitutes a good quality CJ deliberation—we aim to begin to address this gap here.

**Methods:**

We identified the goals that underlie CJs and searched the literature to identify existing frameworks assessing the quality of CJ deliberations. We then mapped the items constituting these frameworks onto the CJ goals; where none of the frameworks addressed one of the CJ goals, we generated additional items that did map onto the goal.

**Results:**

This yielded a single operationalized deductive coding framework, consisting of four deliberation elements and four recommendation elements. The deliberation elements focus on the following: jurors’ preferences and values, engagement with each other, referencing expert information and enrichment of the deliberation. The recommendation elements focus on the following: reaching a clear and identifiable recommendation, whether the recommendation directly addresses the CJ question, justification for the recommendation and adoption of societal (rather than individual) perspective. To explore the alignment between this framework and the goals underlying CJs, we mapped the operationalized framework onto the transcripts of a CJ.

**Conclusion:**

Results suggest that framework items map well onto what transpires in an actual CJ deliberation. Further testing of the validity, generalizability and reliability of the framework is planned.

## 1 INTRODUCTION

Public participation in health policy processes is believed to improve the quality of decision making, enhance the legitimacy of decisions and build capacity among both decision makers and publics.^1^ Citizen/Community Juries (CJs) are a deliberative democratic process and a form of public participation that aims to elicit an informed community perspective on topics that are viewed as controversial or divisive, crowded with voices of those with competing interests or have scientific uncertainty around the balance between benefits and harms.^2^, ^3^

Citizen/Community Jury methods rest on the assumption that an informed public can be brought together to engage in high‐quality dialogue and craft “thoughtful well‐informed solutions”^4^ to difficult problems. In a published essay resulting from a 2‐day symposium of 25 deliberative researchers, Blacksher and colleagues^5^ identified three core elements of public deliberation processes: provision of balanced information; inclusion of diverse perspectives; and reflection and discussion opportunities. If done well, they further suggested three normative goals could be achieved: an informed citizenry; reciprocity and mutual respect; and public‐spirited/“common good” recommendations. Similarly, in their handbook, the Jefferson Centre^4^ proposes that CJs deliver in‐depth learning to participants, respectful and focussed discussions, “common ground solutions,” but also expression of values and concerns, thoughtful and informed input and reasons for their recommendations. Similar elements are noted elsewhere in the literature, including the emphasis on the importance of expression and consideration of participants’ values,^6^, ^7^, ^8^ interactions between participants and exchange of diverse viewpoints,^6^, ^7^, ^8^ the importance of decisions that are better informed^7^, ^8^ and recommendations that address the “common good.”^8^

Reporting standards for CJs have been proposed to increase the transparency and trustworthiness of CJ *processes*.^9^ Although this may help to facilitate the quality of the organization processes (by better reporting standards that aid critical reflection), it does not inform us about whether the goals of the CJ are upheld. That is, it does not inform us about the *content* of the CJ deliberation—about “what happens as people deliberate”.^10^

Some researchers who use deliberative methods have provided tools to assess aspects or elements of the deliberation. This includes, for example, discourse quality of deliberative processes (eg Discourse Quality Index^11^, ^12^) that analyses the speech (or discourse) of the participants. Others still have suggested frameworks that consider the structure of deliberative events, their process and outcomes.^10^, ^13^, ^14^ Despite the growth in scholarship on deliberative methods and an increasing interest among policymakers in the publicly generated evidence that these processes can provide, there are no benchmarks for good quality CJ content, and our understanding of the goals assumed to underpin CJs—such as respect, justified reasoning and turn‐taking—remains limited.

To begin to address these gaps, we developed a deductive qualitative coding framework that operationalized the theoretical goals underlying CJ methodology and deliberations. To explore the utility of this framework, we apply it to transcripts of a recent CJ on dementia. Briefly, the Community Jury on case‐finding for dementia recruited 50‐ to 70‐year‐olds with no previous self‐reported diagnosis of dementia or Alzheimer's disease or mild cognitive impairment. Jurors were randomly selected from the local community using landline‐based sample; quotas were utilized to ensure balance of gender and education. The Jury consisted of 10 participants (five male and five female), whose average age was 62, and educational level was mixed, ranging from some secondary education to university postgraduate. Full details of that CJ have been reported elsewhere.^15^

## 2 METHODS

First, we compiled the “goals” of the community jury deliberation processes from two primary sources: the Citizens Jury Handbook^4^ and an article presented and further developed in a two‐day symposium on public deliberation attended by 25 deliberative researchers.^5^ We chose these sources particularly because one is the published manual which articulates the assumptions/goals of the CJ process and the other represents the deliberation of deliberative researchers. Nevertheless, these “goals” are widely accepted in the literature as conditions that create effective deliberation.^6^, ^7^, ^8^

We then conducted a literature search to identify existing quality frameworks or tools (focused on process and/or content) used in CJs specifically and other deliberative processes more generally. The literature search was not meant to be exhaustive, but rather, to provide illustrative frameworks or instruments or tools (hereafter “frameworks”) from which we could build a deductive qualitative coding framework to analyse CJ deliberations.

Finally, we mapped the frameworks identified in the literature against the goals underlying CJ deliberative methods, to identify whether any of these goals are currently unaddressed by the existing frameworks. Where this was the case, we added an item—this yielded the operationalized deductive coding framework (Table 1). Finally, we mapped the operationalized framework onto the transcripts of deliberations a CJ on dementia to explore the alignment between the framework and the underlying goals of a CJ (Table 2).

**Table 1:** Mapping key CJ goals onto published coding frameworks

Goals of CJ deliberation processes	Frameworks to assess deliberation content					
	De Vries et al^10^	Longstaff & Secko^17^	Anderson & Hansen^16^	Han et al^11^ Himmelroos^12^ Discourse quality index	O'Doherty^14^	Operationalized deductive coding framework
G1: Express values and preferences of participants						Does the CJ deliberation refer to individuals’ values and preferences?
G2: Reciprocal interactions and consideration of alternative views	Participant engagement; Respect; Openness to complexity	Outputs reflected a broad view of the situation that addressed all issues considered important by participants; Participants accepted the assessment or decision process as having conformed to standards of sound analysis and decision making, even if they did not agree with the final assessment or recommendation for action	Increasing mutual understanding among participants; Openness towards the arguments of others	Interactivity (evidence participants are interacting with one another evidenced by referencing names or statements); Constructive politics (degree to which participants offer alternative proposals or attempt to mediate proposals)	Listen to others and take views into consideration	Did the jurors engage with each other's perspectives during the deliberation?
G3: Enhance participants’ knowledge	Use of on‐site experts; Use of incorrect information; Learning new information;	Information was added	Educating citizens			Does the CJ deliberation reference information from the experts?
G4: Produce thoughtful, well‐informed solutions	Understanding and application of information;	More information was considered in the process; Public participants became better informed about relevant environmental, scientific, social and other issues		Consideration of trade‐offs (participants weigh potential advantages and disadvantages of proposals)		Does the information provided by the experts enrich the deliberation? Has the CJ reached a clear and identifiable recommendation? Does the CJ recommendation directly address the charge the CJ was given?
G5: Provide reasons for recommendations	Impact of information on opinions; Justification of opinion;		Formation of reasoned opinion	Justification rationality (extent to which participants offer justifications for their claims) Level and content of justification	Considered opinions offered	Do the jurors provide justification(s) for the recommendation they reached?
G6: Produce recommendations from a societal (rather than individual) perspective	Adoption of societal perspective		Minimizing the use of arguments referring to narrow self‐interest	Common good orientation (extent to which participants express claims that address more collective than personal impacts)	Reach agreement	Does the CJ deliberation reflect a societal perspective?

G, Goal.

**Table 2:** Deductive coding framework analysis

Elements	Operationalized goal	Explanation	Text that could be coded as demonstrating that this element is present
Deliberation Elements	G1: Does the CJ deliberation refer to individuals’ values and preferences?	Do the jurors raise values and preferences (eg autonomy, transparency, greater good, etc) during the deliberation	*“The progression [of dementia] being slow anyway, there's no benefit of finding it early, plus the effect of depression that it would have on the person. But on the yes side […] I guess it would give you time to prepare yourself and your family, to find the best quality of life you can have as the illness progresses […]” (Juror 9, p43)*
	G2: Did the jurors engage with each other's perspectives during the deliberation?	Do the jurors engage with each other's points, views and arguments? (Eg via clarification, agreement, building on each other's point…)	“*I also agree with [Juror 9, who stated her position just prior], 50 does seem a bit young. I think we should look at raising the age. At the same time doing that, making communities in general aware of educational programs through the media” (Juror 7, p44)*.
	G3: Does the CJ deliberation reference information from the experts?	Do the jurors raise (eg cite) points that were made by experts during expert presentations, unprompted by facilitators/note‐takers.	*“… [the] car driver patient, driving the car… and she was a clear and present danger to all by observations of other people in the community, yet there was nothing that she ‐ that the doctor could concretely diagnose her with to get her off the road, to get her the help that she needed […].” (Juror 7, p57)*
	G4: Does the information provided by the experts enrich the deliberation?	Do the jurors engage with the points made by experts during the presentations—for example a juror raises a point, and others engage with it (eg challenge it, affirm it, negate it, provide further clarification or examples pertinent to the point raised, etc)	*Juror 2: Really what we found out yesterday, that the family history is very much non‐existent, that they said it's about 45 families in the whole of Australia that have been identified.* *Juror 5: As hereditary?* *Juror 8: That's only identified that, you know, there's probably a lot more that haven't been identified in the findings.* *Juror 2: Is there family history because of lifestyle?* *Juror 7: No, genetics.* *Juror 8: Genetics.* *Juror 2: Does it say that?* *Juror 5: No but there is ‐ everything's got genetic somewhere.* *Juror 7: That's what family history is.* *Juror 3: Yeah, family history is genetics. (Jurors 2,5,8,7, 3, p96)*
Recommendation Elements	G4: Has the CJ reached a clear and identifiable recommendation?	Do the jurors reach a recommendation that can be clearly identified?	*The jury's recommendation was the following: No, the health system should not encourage GPs to case find (Juror 7, p116*)
	G4: Does the CJ recommendation directly address the charge that the CJ was given?	Do the jurors reach a recommendation that directly responds to the issue or charge or topic that the jury aimed to address?	No, the health system should not encourage GPs to case find for dementia. However, because the jurors recognized that Australian GP Guidelines encourage doctors to practice case‐finding for dementia, they suggested several amendments to the Guidelines—including that the testing be conducted by specialists rather than GPs; there ought not to be financial incentives for testing; education on testing and treatment options should be provided to the community and to health‐care providers; age limit should be removed in favour of eligibility on the basis of risk factors; and several other amendments^22^
	G5: Do the jurors provide justification(s) for the recommendation they reached?	Are reasons offered in support of the recommendation reached?	No effective treatment: *Juror 5: “I think that until there is a definite chance of stopping or fixing the problem, it [case‐finding for dementia] would create a far greater negative outcome than a positive one” (p5)* Case‐finding takes place too early in the course of the disease: *Juror 5: “You know, we're getting told very early when it's going to be 10 years before it appears, that would be 10 ‐ for a lot of people, that would be 10 years of worry.” (p49)*
	G6: Does the CJ deliberation reflect a societal (rather than individual) perspective?	Do the jurors differentiate between the decision they would make for themselves personally and the decision they would make for the community as a whole	*"this [decision] is about the overall good for everybody and we're taking ourselves out of the equation and making a choice that involves ‐ sorry, making a choice that involves everybody" (Juror 7, p43)*

G, Goal.

### 2.1 Stage 1: Developing a deductive qualitative coding framework using existing content assessment frameworks for CJs

We searched PubMed and ProQuest on 8 January 2018 for quality assessment frameworks referencing Community/Citizen's Juries, deliberative democracy and public deliberation. We did not restrict the search by language or date (Appendix 1). As it was a focussed literature search, we chose to search on the term “quality” rather than the broader terms “evaluation” or “assessment,” as “quality” was the term typically used by researchers known to focus on the assessment of public deliberation exercises.^10^, ^11^, ^13^

**Figure 1:**
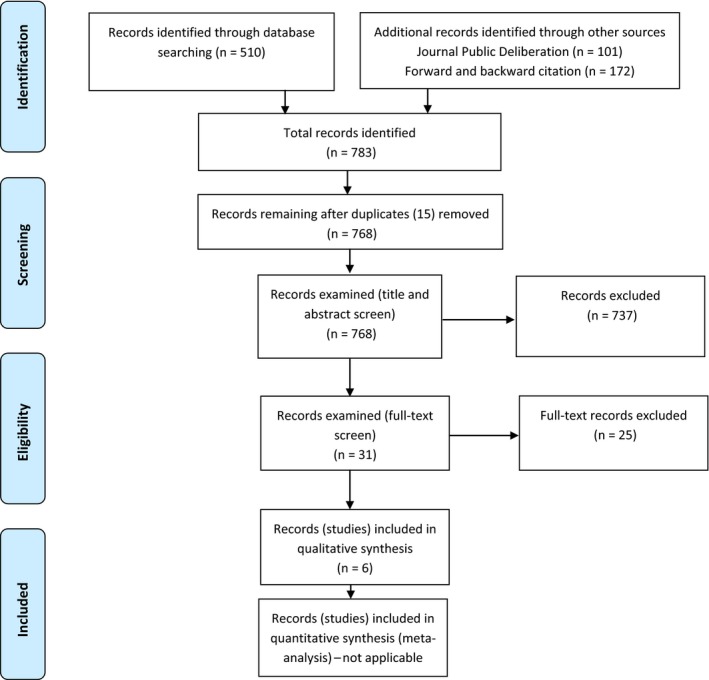
Search results

Two authors independently examined the title and abstract of each reference resulting from the literature searches to identify those articles which discussed quality assessment frameworks applicable to CJs (Figure 1). Where an article's title or abstract suggested that the article did not discuss this, we excluded it from further consideration. Where an article's title or abstract suggested that this article did do so, we read it in full. We then excluded those articles which, upon reading in full, were found not to discuss a framework and included those that did do so. The latter comprises the set of “studies included in qualitative synthesis” (Figure 1).

The institutional websites of all the authors whose articles discussed a framework were also examined to identify any further work on this topic (as indicated by, eg the lists of publications on their websites or a CV). We additionally examined the reference lists of all of the articles that discussed the quality frameworks (backward citation) and searched for articles that subsequently cited our included studies (forward citation). The latter was done on the assumption that any subsequent quality assessment framework on this topic would cite these earlier references. Finally, we also hand searched the contents of the *Journal of Public Deliberation*. All of the steps were conducted independently by two authors, with discrepancies in decisions resolved by consensus.

Items from articles that potentially assessed *content* quality were extracted to form a preliminary list of relevant quality assessment items based on the literature. Finally, we reviewed this list of potential quality assessment frameworks, compared them with the goals of CJs, and operationalized these to form a deductive qualitative coding framework.

### 2.2 Stage 2: Analysing CJ deliberations using the deductive qualitative coding framework

Using the coding framework developed from stage 1, we piloted the application of the framework on transcripts of the jurors’ private deliberations during a recent community jury on case‐finding for dementia^15^ that was conducted in March 2017. We asked participants of this CJ “Should the health system encourage General Practitioners to practice ‘case‐finding’ of dementia in people older than 50?”. Two authors independently examined transcripts of CJ discussions from day 2 using the deductive qualitative coding framework to identify whether text that supported the presence of the framework's elements can be identified.

## 3 RESULTS

The results of the literature searches were amalgamated, and duplicate references removed, leaving 768 references to examine. On examination of those references’ titles and abstracts, 737 references were excluded from further consideration as they did not discuss a quality assessment framework. We read 31 references in full text, excluding 25 of them for failing to discuss a quality assessment framework and including six that did do so (see Figure 1 for search results). We mapped each of the five frameworks (Discourse Quality Index was used in two articles) onto the goals of the community jury deliberative process, as identified in the literature.^4^, ^5^, ^6^, ^7^, ^8^ Table 1 maps the quality frameworks described in the included papers to key goals of public deliberation and CJs. Table 2 explores the utility of the proposed framework.

### 3.1 Goal 1: Express values and preferences of participants

The Citizens Jury Handbook stipulates a Citizens’ Jury is an opportunity for policymakers and decision makers to “learn about the values, concerns and preferences of the community members”.^4^

None of the included frameworks have explicitly incorporated this goal. This was therefore operationalized by asking the following question: “Does the CJ deliberation refer to individuals’ values and preferences?” This queries whether jurors made explicit references to values and preferences during the deliberation—for example, by referencing issues such as the value of knowing (or conversely, not knowing) a particular piece of information, as well as autonomy, transparency, the greater good and so on.

*Applying the framework*: Participants in the case‐finding for dementia CJ verbalized values and preferences throughout the deliberation transcripts. Participants spoke of preferences and values of obligation towards patients and their families, towards communities regarding further education, potential costs to the health system and professional responsibilities of general practitioners. For example, in response to a discussion about a potential 10‐year time period between case‐finding of diagnosis and progression of dementia, a juror noted:

(Juror 5) "for a lot of people, that would be 10 years of worry… unnecessary worry and concern, and for their families". (p49: psychological impact on patient and families)

### 3.2 Goal 2: Reciprocal interactions and consideration of alternative views

Respectful and reciprocal discussions between the jurors are frequently recognized as a key goal of the community jury process.^4^ Blacksher further argues that in a public deliberation, jurors should be able to have an opportunity to engage with each other to “articulate and justify their positions and weigh alternate views.”^5^

That is, a CJ should not only offer an opportunity for jurors to voice their own opinions—but also to consider and learn from the opinions of others, whether they agree or disagree with those opinions.

This goal is frequently reflected in the included frameworks. For example, Anderson and Hansen^16^ frame this as “increasing mutual understanding among participants” and “openness towards the argument of others”; and O'Doherty^14^ as listening to other jurors and taking their views into consideration. Somewhat more implicitly, Longstaff and Secko^17^ emphasize that outputs of the community jury should reflect “a broad view of the situation that addressed all issues considered important by participants,” and Han^11^ and Himmelroos^12^ also raise the importance of interactivity between participants. We operationalized this as 'Did the jurors engage with each other’s perspectives during the deliberation?'

*Applying the framework:* The engagement with each other's views and reciprocal interactions were both evident throughout the CJ process. The following exchange reflects an engagement and reciprocal interaction via a request for clarification of views:

(Juror 8) Could I clarify one thing on that? (Juror 4) Oh sorry. (Juror 8) Could I clarify one thing on that? (Juror 4) Sure. (Juror 8) By what you said, is it more a case of they only do the case‐finding if there's symptoms or when they do the 50 wellness check, they do everybody? (Juror 4) No, it's only people ‐ not a broad screening of everyone, it's just if someone comes in…(Juror 2) If requested. (p41)

### 3.3 Goal 3: Enhance participants’ knowledge

At a minimum, public deliberation has been argued to be “based on balanced factual information that improves [participants’] understanding of a topic… [and] leave citizens better informed about the issue”.^4^, ^5^ Consequently, as a form of public deliberation, a key aim of a CJ is to arrive at an *informed* decision often achieved through presentations that aim to compliment or enhance jurors’ knowledge on the topic.

The importance of this element is recognized in several of the identified frameworks. For example, De Vries et al^10^ recognize its importance by identifying both the use of on‐site experts and learning new information as important elements of their framework; in a similar vein, Anderson and Hansen^16^ highlight “educating citizens” and Longstaff and Secko emphasize that information be added as part of the CJ.^17^

In the proposed coding framework, this element is operationalized by the following: “Does the CJ deliberation reference information from the experts?” This questions whether the jurors raised (for example by citing or paraphrasing) points from the expert presentations without prompting by the facilitators.

*Applying the framework*: Throughout the deliberation, participants in the CJ referenced information provided by the experts. For example, a specific example offered by an expert was considered:

(Juror 5) “But like that lady doctor said yesterday, there are a percentage of things that happen where families have wanted them to be declared with Alzheimer's when they might not be so they can get hold of their house and sell it and whatever, whatever”. (p106)

Jurors also helped each other to understand the information provided by the experts:

(Juror 5) “Didn't the doctor say when we asked him about the education, did he say that they had found that people who have a lesser education were inclined to get this, or did he…” (Juror 7) “There was a higher incidence, yes”. (p100)

### 3.4 Goal 4: Produce thoughtful, well‐informed solutions

Community juries’ goal is to be an effective means of developing a solution to a problem or an issue that is thoughtful and well informed.^4^ Two key dimensions constitute this goal: the thoughtful and well‐informed aspect, and the solution aspect.

First, the thoughtful and well‐informed dimension suggests that community jurors will go beyond exposure to new knowledge and its repetition to actively engaging with the new knowledge. Several of the frameworks include this element, highlighting the “understanding and application of information,”^10^ that additional (new) information be considered in the community jury process^17^ and that jurors carefully weigh both the advantages and disadvantages of proposals being considered.^11^, ^12^ To guide our coding, we operationalized this as, “Does the information provided by the experts enrich the deliberation?”. The notion of enriching is deployed here to signal going beyond mere reiteration of experts’ points to active engagement with them—for example by challenging, affirming, negating and clarifying.

*Applying the framework*: Numerous examples of juror dialogue during the deliberations reflect this. For example, this discussion about the prevalence of hereditary dementia in Australia, which was raised in one of the expert presentations:

(Juror 2) “Really what we found out yesterday, that the family history is very much non‐existent, that they said it's about 45 families in the whole of Australia that have been identified.” (Juror 5) “As hereditary?” (Juror 8) “That's only identified that, you know, there's probably a lot more that haven't been identified in the findings.” (Juror 2) “Is there family history because of lifestyle?” (Juror 7) “No, genetics.” (Juror 8) “Genetics.” (Juror 2) “Does it say that?” (Juror 5) “No but there is ‐ everything's got genetic somewhere.” (Juror 7) “That's what family history is.” (Juror 3) “Yeah, family history is genetics”. (p96)

In addition to being well informed, CJs aim to produce a recommendation (potential solution) that actually addresses the charge or question issued to the community jury. We did not identify items from other frameworks that specifically address this goal. We operationalized this using two coding questions: “Has the CJ reached a clear and identifiable recommendation?” and “Does the CJ recommendation directly address the charge that the CJ was given?” The first asks whether the jury reached *any identifiable* recommendation that can be discerned from its transcript, as opposed to failing to come to a recommendation. The second queries whether the jurors’ recommendation *specifically addresses* the specific charge or challenge that was issued to the jury.

*Applying the framework* **: **The jury recommendation was clear and identifiable in the deliberation transcript, and it directly addressed the charge that the jury. The jurors unanimously voted against the jury charge: “Should the health system encourage GPs to practice case‐finding of dementia in people older than 50?” clearly and identifiably offering a recommendation (against the practice of case‐finding in dementia), and directly addressing the question. In addition to this, the jury also recognized the practice was currently endorsed and so also made some recommended amendments to the current guidelines.

(Juror 7)…and it's [the existing guideline] probably here to stay. So given that it's here to stay, we'd like to adjust these guidelines and the last part we were discussing was to do with referring to specialists, rather than the GP and early intervention and prevention, that there should be awareness and education out there in the public sector for everybody to make their own choices. (p118)

### 3.5 Goal 5: Provide reasons for recommendations

Community juries need to not only produce a recommendation—but one that is backed by reasons or justifications.^4^, ^5^ This goal recurs throughout the frameworks identified in the literature. For example, De Vries et al stress the import of “justification of opinion,”^10^ and Anderson and Hansen^16^ emphasize the formation of opinions that are reasoned. Similarly, O'Doherty notes that opinions offered ought to be “considered.^14^” In the proposed coding framework, this element is operationalized by the following: “Do the jurors provide justification(s) for the recommendation they reached?” Quotes to assess this can be drawn from those parts of the transcript where the jurors enunciate their reasons for their judgement for or against the recommendation.

*Applying the framework*: The community jurors offered a wide range of reasons for their recommendation against case‐finding for dementia. These included the following: the absence of effective treatments, timing in the course of disease, impact of the results on the individual's mental and emotional health, and others.^15^ For example:

Juror 10: “I see that to be diagnosed and told that you are destined to become a person with dementia, will be devastating for anyone. For those patients who are misdiagnosed and caused unnecessary fear and indignity, it would be far worse”. (p45)

### 3.6 Goal 6: Produce recommendations from a societal (rather than individual) perspective

Community juries bring community members together to answer an issue or challenge *not* from a personal perspective (what the juror would do individually) but rather from a community perspective (what we as a community would like to do collectively).^4^, ^5^

This goal is commonly reflected in the frameworks. For example, De Vries^10^ notes the “adoption of societal perspective,” and similarly, Han^11^ and Himmelroos^12^ emphasize the “common good orientation.” Anderson and Hansen^16^ approach the issue from the opposite side, noting the importance of “minimizing the use of arguments referring to narrow self‐interest.” We operationalized this goal by querying: “Does the CJ deliberation reflect a societal (rather than individual) perspective?” Is there evidence in the transcripts that the jurors differentiated between the decision they might make for themselves personally and the decision they would make for the community as a whole.

*Applying the framework*: This goal is exemplified in the following juror remarks:

(Juror 4) "So I'm a yes for that one, it should be done. It's different to my opinion on individual, because would I go and do it, no". (p41)

(Juror 7) “So that's what this is about, looking for case‐finding, the benefits outweighing the harms or vice versa, for the majority of people, not just how we look at it in our own mindset”. (p55)

## 4 DISCUSSION

To our knowledge, this is the first attempt at generating a deductive coding framework and mapping it against the key goals of Citizen/Community Jury by analysing the transcripts of CJ deliberations. As CJs are becoming more popular for addressing complex policy questions in a wide range of areas,^18^ it is becoming increasingly important to ensure the CJs meet the goals of a deliberative democratic process, such as participant engagement and reciprocity, expression of preference and values, and well‐informed recommendations.

We identified six key goals of public deliberations and Citizen/Community Juries more explicitly.^4^, ^5^ The proposed coding framework addresses these, by using eight questions which are directly mapped to quality frameworks identified from the literature. The proposed framework brings together these goals and quality frameworks and operationalizes them by developing questions to help guide analyses of deliberative transcripts. The coding framework has the potential to improve the use of CJs by demonstrating their capacity to uphold the goals of deliberative processes to produce considered and informed recommendations for the society as a whole. Our coding framework can be used retrospectively and prospectively. Retrospectively, it can assess whether the goals of deliberative processes were met, while prospectively, it can help to guide the facilitator to structure deliberation to meet the goals of CJs.

Lack of uptake of the recommendation generated from past CJs indicates that policymakers may lack trust in CJ processes—or that CJ sponsors (researchers, policymakers, etc) are not building their translation processes into CJ design.^19^ Researchers have conducted multiple CJs, experimented with methods (eg recruitment, presentations of experts, dissemination of materials and quantitative analyses of knowledge)^18^, ^20^ and written reporting templates in an attempt to provide evidence of robustness and stability of decision making.^9^, ^21^ The approach proposed here is an attempt to explore whether CJ deliberations uphold the goals of deliberative process and thus provide another reason for decision makers to trust the outcome of CJ processes.

It is a potential limitation that the six goals considered here have been derived from two key documents,^4^, ^5^ as this leaves open the possibility that additional goals, considered elsewhere in the literature, may have been missed. The comprehensiveness of these six goals will therefore have to be formally corroborated. However, it is reassuring that most of the six goals considered here are echoed elsewhere in the literature on the theoretical goals that underpin community jury and deliberative democracy approaches more generally.^6^, ^7^, ^8^ Likewise, it is a potential limitation that in developing this coding framework, we have also explored its use in one CJ. We welcome other CJ researchers to use this framework to assess its validity, generalizability and reliability. We have planned a second pilot evaluation to compare the researchers’ qualitative assessment of each of the framework's items from the CJ deliberative transcript, with the self‐reported views of the jurors using survey items which were designed to align with the framework.

To improve the trustworthiness of CJs in the minds of policymakers, we must demonstrate that the constructs of robust deliberative democratic techniques are upheld. This coding framework has the potential to assess CJ deliberations at least as they pertain to the key goals of Citizen/Community Juries and deliberative democratic processes. Used together with the CJCheck reporting template^9^ to describe CJ processes, we can progress towards routinely using deliberative democratic techniques like CJs for difficult and controversial health policy decision.

## CONFLICT OF INTEREST

All of the authors (AMS, RS, CD, SC, RT) have been previously involved in organizing Citizen/Community Juries. AMS, RS, and RT organized the Dementia Community Jury specifically referred to in the manuscript. AMS salary is funded by an NHMRC Centre for Research Excellence grant (1044904). RS and RT are funded by an NHMRC Program grant (1113532). SC and CD are funded by an NHMRC research grant, 1104136. CD is also funded by NHMRC project grant, 1083079. The funding bodies had no involvement in study design; in the collection, analysis and interpretation of data; in the writing of the articles; and in the decision to submit it for publication.

## ETHICS APPROVAL

Ethics approval for this project (Deductive Coding Framework) was not sought as no human subjects were involved in the research. The original project (Dementia Community Jury) sought and received ethics approval from the Bond University Human Research Ethics Committee (approval # 15810).

## APPENDIX 1

### LITERATURE SEARCHES

1. Search of the PubMed database:

Search string: ((((deliberative democra*[Title/Abstract] AND quality[Title/Abstract])) OR ((community jur*[Title/Abstract]) AND quality[Title/Abstract])) OR ((citizen* jur*[Title/Abstract]) AND quality[Title/Abstract])) OR ((public deliberat*[Title/Abstract]) AND quality[Title/Abstract])

2. Search of the ProQuest database

Search string: AB("deliberative democral*" OR "community jur*" OR "citizen* jur*" OR "public deliberat*") AND quality

3. Hand search of the contents of the *Journal of Public Deliberation*
4. Forward (cited by) and backward (citing) citation searches of the included articles.
